# Image guidance and interfractional anatomical variation in paediatric abdominal radiotherapy

**DOI:** 10.1259/bjr.20230058

**Published:** 2023-05-10

**Authors:** Sabrina Taylor, Pei Lim, Jessica Cantwell, Derek D’Souza, Syed Moinuddin, Yen-Ching Chang, Mark N Gaze, Jennifer Gains, Catarina Veiga

**Affiliations:** 1 University College London, Centre for Medical Image Computing, London, United Kingdom; 2 Department of Oncology, University College London Hospitals NHS Foundation Trust, London, United Kingdom; 3 Radiotherapy, University College London Hospitals NHS Foundation Trust, London, United Kingdom

## Abstract

**Objectives:**

To identify variables predicting interfractional anatomical variations measured with cone-beam CT (CBCT) throughout abdominal paediatric radiotherapy, and to assess the potential of surface-guided radiotherapy (SGRT) to monitor these changes.

**Methods:**

Metrics of variation in gastrointestinal (GI) gas volume and separation of the body contour and abdominal wall were calculated from 21 planning CTs and 77 weekly CBCTs for 21 abdominal neuroblastoma patients (median 4 years, range: 2 – 19 years). Age, sex, feeding tubes, and general anaesthesia (GA) were explored as predictive variables for anatomical variation. Furthermore, GI gas variation was correlated with changes in body and abdominal wall separation, as well as simulated SGRT metrics of translational and rotational corrections between CT/CBCT.

**Results:**

GI gas volumes varied 74 ± 54 ml across all scans, while body and abdominal wall separation varied 2.0 ± 0.7 mm and 4.1 ± 1.5 mm from planning, respectively. Patients < 3.5 years (*p* = 0.04) and treated under GA (*p* < 0.01) experienced greater GI gas variation; GA was the strongest predictor in multivariate analysis (*p* < 0.01). Absence of feeding tubes was linked to greater body contour variation (*p* = 0.03). GI gas variation correlated with body (*R* = 0.53) and abdominal wall (*R* = 0.63) changes. The strongest correlations with SGRT metrics were found for anterior-posterior translation (*R* = 0.65) and rotation of the left-right axis (*R* = −0.36).

**Conclusions:**

Young age, GA, and absence of feeding tubes were linked to stronger interfractional anatomical variation and are likely indicative of patients benefiting from adaptive/robust planning pathways. Our data suggest a role for SGRT to inform the need for CBCT at each treatment fraction in this patient group.

**Advances in knowledge:**

This is the first study to suggest the potential role of SGRT for the management of internal interfractional anatomical variation in paediatric abdominal radiotherapy.

## Introduction

Neuroblastoma accounts for around 6% of all paediatric cancers in the UK, and radiotherapy plays a pivotal role in the multimodal treatment pathway for high-risk and some intermediate-risk patients.^1–4^ Radiotherapy starts with the acquisition of a planning computed tomography (CT) scan for delineation of both target volumes and organs-at-risk, followed by dosimetric planning and treatment optimisation. The optimised radiotherapy plan is then delivered fractionated over several weeks of treatment. However, the planning CT represents a snapshot of the patient’s anatomy at a specific point in time and the internal anatomy at each treatment fraction may vary due to day-to-day changes in organ filling, body weight, and tumour size, amongst other reasons.^5,6^ Approximately 80% of neuroblastoma tumours are located within the abdomen,^7^ and this part of the body is susceptible to anatomical variations due to the highly variable lumen contents in the gastrointestinal (GI) tract.^8^ This may compromise the conformality of the dose distributions delivered, leading to tumour underdosage (potentially resulting in increased risk of recurrence) or overdosage of normal tissues (potentially resulting in excessive toxicity).^8,9^ Accurate treatment delivery is particularly important for high-risk neuroblastoma patients given that the 5-year overall survival rate remains ~50%.^4,10^

The growing use of highly conformal radiotherapy modalities aiming to improve outcomes in high-risk paediatric abdominal neuroblastoma, such as intensity modulated arc therapy (IMAT) and proton beam therapy (PBT),^9,11^ make it increasingly important to monitor and account for anatomical change. PBT is an attractive treatment option for children due to its better tissue-sparing capabilities, but variations in the tissue density and composition may distort these desirable dose distributions. GI gas volume has been reported to vary as much as 80% during treatment compared to planning CT in pancreatic cancer radiotherapy plans^12^ and this variation has been linked with proton dose degradation in cervical, gastric and pancreatic cancer patients.^8,12–15^ Given that treatment pathways for adults differ greatly from children, there is a knowledge gap where findings on interfractional observations may not be accurately extrapolated to inform paediatric radiotherapy plans. Only a few studies have focused exclusively on interfractional variations in paediatric abdominal radiotherapy with assessment of bowel variation still being poorly investigated.^16–18^ Lim et al^9^ reported that GI gas variation may compromise PBT dosimetry in children with high-risk midline neuroblastoma, reporting possible loss of the clinical target volume coverage up to 15.7% (compared to 1.9% for IMAT). Definite conclusions on the effect of anatomical variation on paediatric PBT dosimetry however are limited by the small sample sizes.^9,19^

Image-guided radiotherapy (IGRT) technologies, such as cone-beam-CT (CBCT), can identify three-dimensional anatomical variations, which provides the opportunity to review the dosimetry and adapt the plan if needed (online or offline). However, CBCT is associated with dose exposures which are of concern in paediatric radiotherapy patients given the risk of young children developing radiation-induced second malignant neoplasms later in life.^20–22^ Considering imaging exposure in children is particularly important in the era of volumetric IGRT.^23^ Daily CBCT imaging doses are small (3 – 9 cGy and 9 – 29 cGy per CBCT scan for soft tissue and bones, respectively, estimated on a 31 month abdominal paediatric phantom^24^) in comparison with the total therapeutic dose levels, but the cumulative dose of using daily CBCTs over all treatment fractions is of similar magnitude to the typical prescribed doses per fraction. For reference, currently radiotherapy is delivered in high-risk neuroblastoma in 1.5 – 1.8 Gy/fraction, up to approximately 21 Gy or 36 Gy (the latter being currently explored in on-going trials for patients with residual disease at the primary site after surgery).^25–27^ Surface-guided radiotherapy (SGRT) offers an attractive solution to complement current IGRT protocols by tracking the patient’s skin surface. While SGRT has mostly been used to simplify patient setup protocols, there is a potential that surface images may also be able to detect internal anatomical variations and trigger adaptive radiotherapy pathways, although this potential has not yet been demonstrated in paediatric abdominal treatments.^28–30^

A greater understanding of the degree and risk factors associated with abdominal anatomical changes during paediatric radiotherapy could inform optimal radiotherapy modality selection and the development of IGRT protocols and adaptive treatment pathways tailored to each abdominal neuroblastoma paediatric patient. Thus, this study aims to identify patient variables predicting interfractional anatomical variations for paediatric abdominal radiotherapy and to explore the potential of SGRT to detect and measure these changes. This study builds up from an exploratory analysis presented by Lim et al,^9^ where it was suggested that patient variables such as the use of general anaesthesia (GA) during radiotherapy may be associated with greater inter-fractional GI gas variation. Here we considerably expanded this preliminary analysis to include a larger dataset (*n* = 21 vs *n* = 11), and more comprehensively explore image-based metrics of interfractional variation (such as body and abdominal wall separation changes) and patient variables (such as the use of feeding tubes). To the best of our knowledge, this is the first study using volumetric imaging to quantify and to identify potential predictors interfractional anatomical change focusing on high-risk neuroblastoma paediatric patients, while exploring the novel use of SGRT technologies for its detection.

## Methods and materials

This study included data from 21 paediatric patients with high-risk abdominal neuroblastoma historically treated with external beam radiotherapy. Patient characteristics are shown in Table 1. Patients did not receive concurrent chemotherapy. No dietary preparation was given prior to planning or treatment, and patients treated under GA received the same instructions for fasting for planning and treatment. The data for this study were requested and approved in line with the internal information governance procedures of the University College London Hospitals NHS Foundation Trust Radiotherapy Department and provided anonymised.

**Table 1.** Patient characteristics.

Patient characteristics	*N* = 21
**Age (years**)	
Median	4
Mean (Range)	5 (2 – 19)
**Ratio (no.)**	
Male : Female	10 : 11
General anaesthesia (GA) : No GA	11 : 10
Feeding tube : No feeding tube Nasogastric tube : Percutaneous endoscopic gastrostomy	12 : 9 9 : 3

### Imaging scans and segmentations

All patients had one CT for treatment planning purposes and up to five weekly CBCTs acquired during treatment. A total of 21 CTs and 77 CBCTs were analysed and segmented for GI gas and body volumes. Segmentations were carried out semi-automatically using ITK-SNAP (Version 3.8.0).^31^ All contours were automatically post-processed to remove common manual segmentation errors. To define a common field-of-view between the two modalities, CTs and CBCTs were rigidly co-registered using the open-source image registration algorithm NiftyReg.^32^

### Metrics

**Figure 1.**
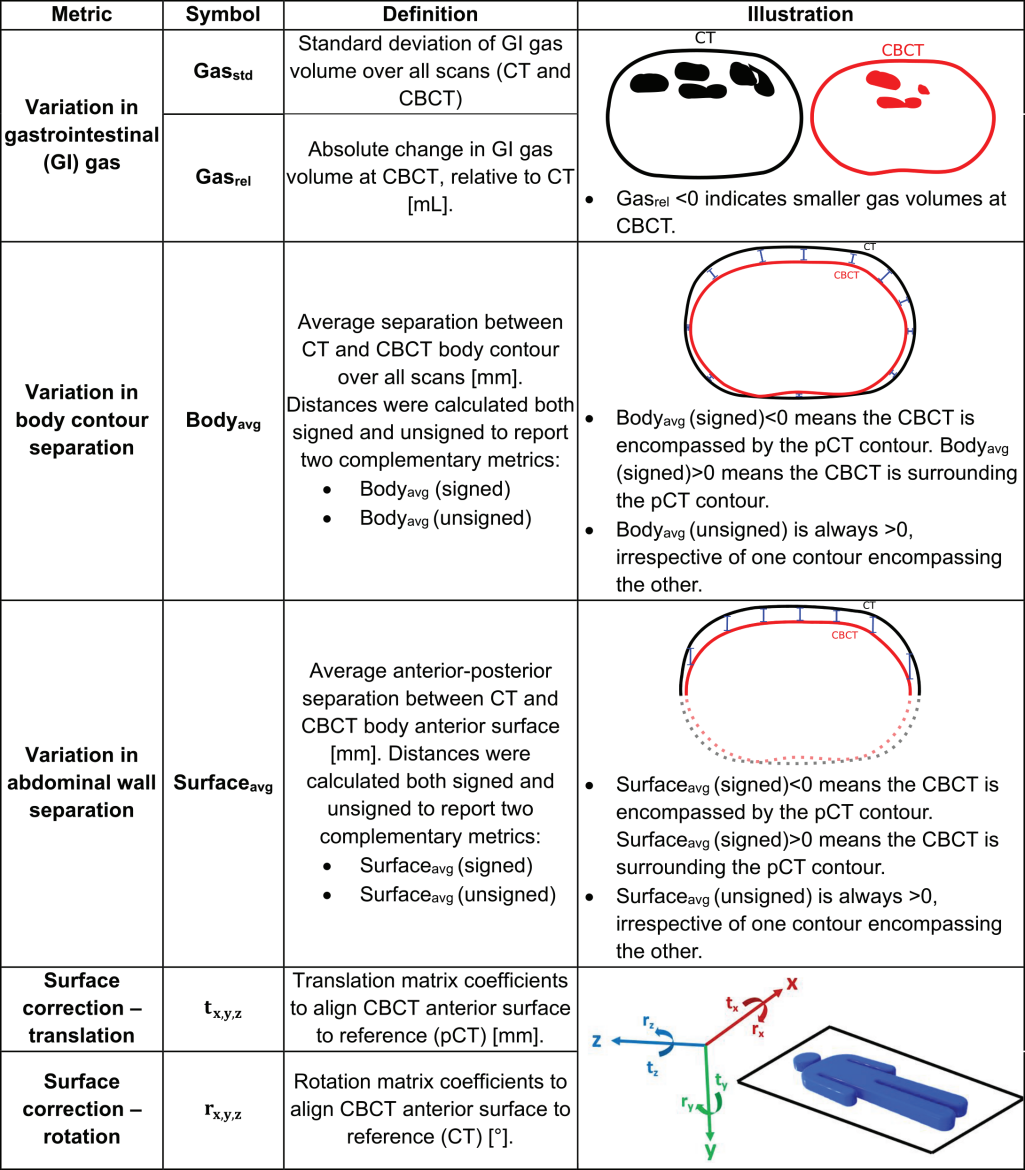
Definition of the metrics of interfractional anatomical change and surface correction between planning computed tomography (CT) and cone-beam CT (CBCT).

GI gas variation and weight changes are types of anatomical change frequently observed in the abdominal region.These variations were measured from the CT and CBCT segmentations, and converted to quantitative metrics as defined in Figure 1.

GI gas volumes were measured from the GI gas segmentations, from which we calculated the standard deviation of the GI volumes across all imaging timepoints (Gas_std_ [ml]), a measure of GI gas variability, as well as the absolute GI volume changes relative to the planning volumes (Gas_rel_ [ml]).

Changes to the body contour may be linked to both GI gas variation and weight loss. The abdominal wall adapts to the internal contents in the gut, such that abdominal distension is related to the volume of gas within the digestive tract.^33^ Thus, we measured both variation in the whole-body contour and at the anterior surface of the body (surrogate for the abdominal wall) to decouple the effects of weight from GI changes. The closest distance between each voxel on the CT and CBCT body contour surface was calculated to generate a distance distribution (bi-directionally). The distributions were then used to calculate two complementary metrics: the signed and unsigned average distance (Body_avg_(signed) and Body_avg_(unsigned) [mm]). Unsigned distance metrics only measure the amount of the anatomical change, not the direction of the change – *i.e.,* by how much the body contour has changed, but not if it shrank or expanded. Positive and negative (signed) distances allow visualisation of the relative position of the contours. For example, a negative signed Body_avg_(signed) indicates the CBCT is encompassed by the CT contour. To quantify changes at the abdominal wall, the signed and unsigned anterior-posterior distance between body contours at the anterior surface only was also calculated (Surface_avg_(signed) and Surface_avg_(unsigned) [mm]).

Finally, surface correction metrics were calculated from the body contours to reflect the correction that a SGRT system would have obtained between planning and treatment position. The treatment position was simulated by applying the previously described six degree-of-freedom transformation to align the CBCT with the CT, followed by applying a translation to both scans such that their origins matched the radiotherapy treatment isocenter. The anterior surface was extracted from the body contours and converted to a set of points in space (point cloud). Point clouds were then registered using the iterative closest point algorithm in MATLAB 2019a (MathWorks Inc) to estimate the residual translational (t_x,y,z_ [mm]) and rotational (r_x,y,z_ [°]) corrections needed to align the CBCT surface to the reference (CT). This was done to investigate SGRT for interfractional anatomical monitoring, rather than setup or intrafractional motion monitoring.

### Experimental design and statistical analysis

Two experiments were designed: (1) to identify variables predicting greater interfractional anatomical variations, and (2) to explore the correlation between volumetric and surface metrics of anatomical change. Sex, age, GA, and feeding tubes were explored as predictive variables for Gas_std_, Body_avg_(unsigned) and Surface_avg_(unsigned). Age groups were defined by splitting the cohort into two: those aged <3.5 (*n* = 8) and ≥3.5 (*n* = 13) years. The volumetric and surface anatomical change metrics correlated were described in Figure 1. Statistical analyses were performed using Stata^®^ MP Version 17.0 (StataCorpLLC) and Matlab 2019a. Statistical significance was assumed when *p* < 0.05 . A sensitivity analysis was conducted in all experiments by excluding the planning CT from the metrics calculation and, when applicable, defining as reference one of the CBCT scans randomly selected.

**Figure 2.**
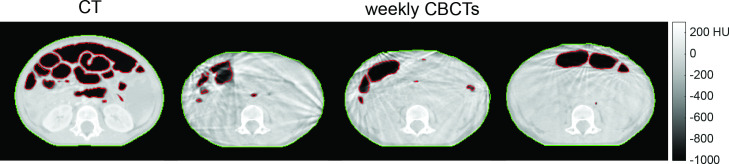
Example of variability in gastrointestinal gas and body contour between planning computed tomography (CT) and multiple weekly cone beam CT (CBCT) scans (Gas_std_ = 171 ml for this subject).

## Results

### Investigation of patient variables predictive of anatomical change

Gas_std_, Body_avg_(unsigned) and Surface_avg_(unsigned) were on average 74 ± 54 ml (range: 5 – 180 ml), 2.0 ± 0.7 mm (range: 0.9 – 3.6 mm) and 4.1 ± 1.5 mm (range: 2.0 – 8.0 mm) throughout treatment across all patients. Patients exhibited on average a trend of reduction in GI gas, body contour and anterior surface across all CBCTs reviewed compared to the planning CT (76%, 86%, and 90% of the patient group, respectively). GI gas variation seen throughout treatment is exemplified in Figure 2.

**Figure 3.**
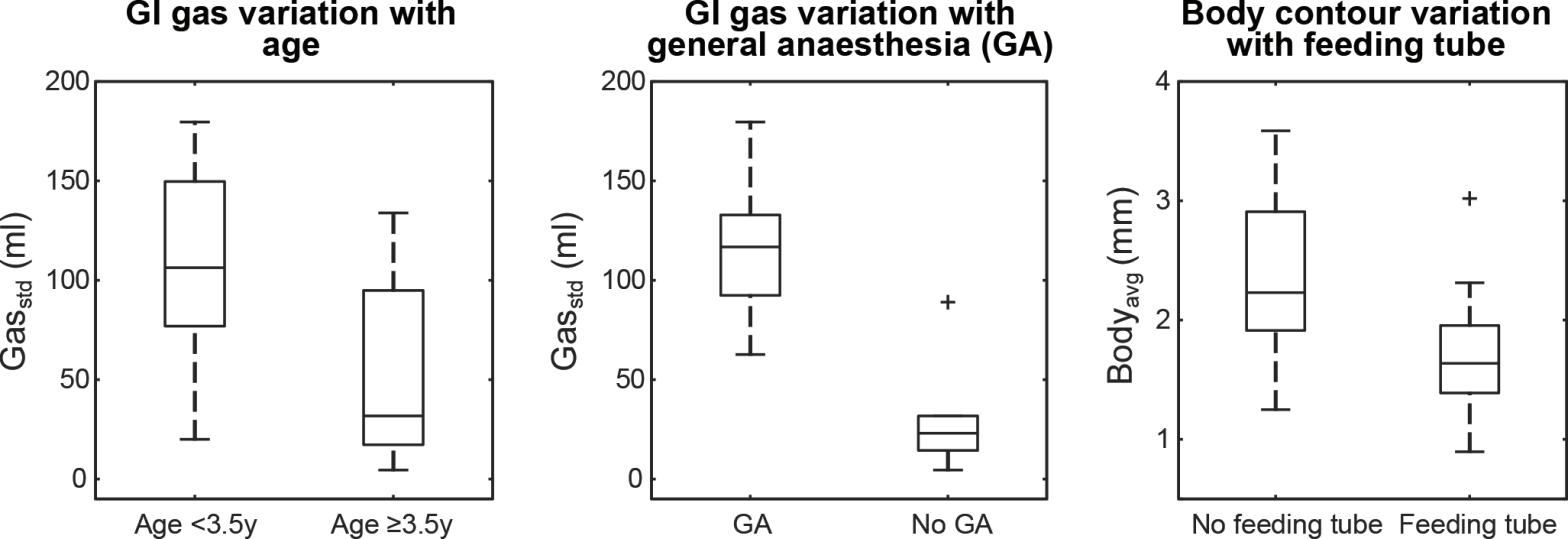
Boxplots of gastrointestinal (GI) gas volume (Gas_std_) variation according to age and general anaesthesia (GA), and body contour variation (Body_avg_) according to absence or presence of feeding tubes. Outliers represent values outside 1.5x the interquartile range.

Gas_std_ was greater for subjects < 3.5 years (*p* = 0.04) and under GA (*p* < 0.01); Body_avg_(unsigned) was greater in patients without feeding tubes (*p* = 0.03) (Figure 3). No variables predicted for Surface_avg_(unsigned). No additional variables predicted for Gas_std_ or Body_avg_(unsigned). All results are summarised in Table 2.

**Table 2.** Statistical analysis for variables predicting anatomical change.

Variables	P-value Mann-Whitney two-sample test		
	**Gas_std_**	**Body_avg_(unsigned**)	**Surface_avg_(unsigned**)
Sex	0.439 (0.622)^*c*^	0.526 (0.622)^*c*^	0.526 (0.622)^*c*^
Age	0.043^*a*^ (0.014^*a*^)^*c*^	0.717 (0.612)^*c*^	0.717 (0.828)^*c*^
General anaesthesia	<0.001^*b*^ (<0.001^*b*^)^*c*^	0.231 (0.159)^*c*^	0.573 (0.260)^*c*^
Feeding tube	0.155 (0.201)^*c*^	0.033^*a*^ (0.434)^*c*^	0.055 (0.356)^*c*^

a *p* < 0.05

b *p* < 0.01

c the values in parenthesis indicate the results when excluding the planning CT scan from the analysis

Statistically significant associations were established between (i) age and Gas_std_, (ii) GA and Gas_std_, and (iii) GA and age (Table 3). Multivariate linear regression analyses highlighted GA as the strongest driver for GI gas variation (*p* < 0.01). Most patients aged <5.5 years received treatment under GA (65%), whereas no patients aged ≥5.5 years were anaesthetised. Only one patient aged <3.5 years did not receive GA.

**Table 3.** Correlation coefficient between patient variables and gastrointestinal gas variation.

Variables	Correlation coefficient	*P* value
Age and Gas_std_	R^*a*^ = −0.573 (−0.683)^*d*^	0.007^*c*^ (<0.001^*c*^)^*d*^
General Anaesthesia and Gas_std_	Coef^*b*^ = 0.069 (0.225)^*d*^	<0.001^*c*^ (<0.001^*c*^)^*d*^
General Anaesthesia and age	Coef^*b*^ = −1.411	0.001^*c*^

a R, Spearman’s rank correlation test coefficient

b Coef, exact logistic regression coefficient

c *p* < 0.01

d the values in parenthesis indicate the results when excluding the planning CT scan from the analysis

All findings remained valid when excluding the planning CT from analysis, only with the exception of the link between Body_avg_(unsigned) and feeding tubes (*p* = 0.43) (Tables 2 and 3).

### Correlations between volumetric and surface metrics of anatomical change

**Figure 4.**
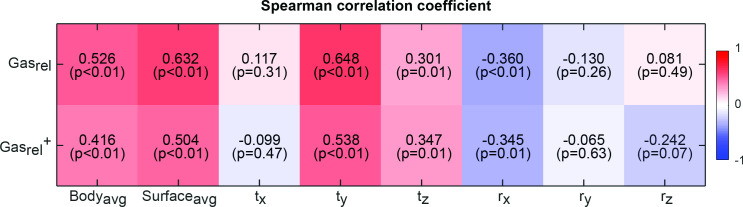
Correlation between gastrointestinal gas variation (Gas_rel_) and metrics of body change (Body_avg_), abdominal wall change (Surface_avg_) and surface correction metrics (t_x,y,z_ and r_x,y,z_). Gas_rel_^+^ indicates the correlations when the CT scan was excluded from the analysis.

**Figure 5.**
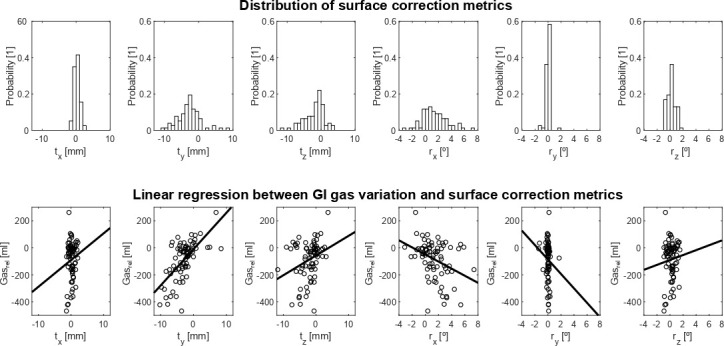
**a**) Distribution of values measured for surface correction metrics (t_x,y,z_ and r_x,y,z_) and b) linear regression with gastrointestinal gas variation (Gas_rel_).

Gas_rel_ was on average −86 ± 138 ml (range: −468 – 262 ml). The signed separation of body contour (Body_avg_(signed)) and body surface (Surface_avg_(signed)) correlated moderately with Gas_rel_ (Figure 4), indicating a link between reduction in GI gas and shrinking of the body contours. Gas_rel_ was more strongly correlated with Surface_avg_(signed) (*R* = 0.63) than with Body_avg_(signed) (*R* = 0.53). Regarding metrics of surface correction, the strongest correlation with Gas_rel_ was found with anterior-posterior translation (t_y_, *R* = 0.65) and rotation of the left-right axis (r_x_, *R* = −0.36). Similar correlations were found when excluding the planning CT from analysis. This suggests that anterior surface changes were more likely affected by abdominal distension driven by GI gas variation, while body contour changes were more likely affected by other interfractional variations, such as weight fluctuation and setup errors. Figure 5 shows the distribution values for each surface correction metric and their linear regression with Gas_rel_. The ranges of values for t_y_ (−2.8 ± 3.3 mm, range: −10.2 – 8.4 mm) and r_x_ (1.4 ± 1.9°, range: −3.1 – 7.4°) are larger than the accuracy reported for commercial surface imaging systems (0.2 mm/0.2°).^28^

## Discussion

This study found that children receiving radiotherapy were more prone to interfractional anatomical variations if they were younger than 3.5 years old, were treated under GA, or were not using a feeding tube. These findings may contribute to inform the selection of the best treatment modality for each patient, such as selecting IMAT versus PBT, and to identify cases benefiting from robust planning pathways and more frequent image-guided protocols to minimise dosimetric inaccuracies. Incorporating SGRT as a key part of clinical IGRT protocols has great promise in childhood cancer radiotherapy where a culture of gentle IGRT is desirable,^23^ with benefits including lower radiation doses and simplified workflows regarding immobilisation and anaesthesia needs. Clinical experience in abdominal paediatric treatments highlighted challenges in the use of SGRT for positioning due to changes in the abdominal wall caused by bloating or constipation.^28^ The established correlation between GI gas and body contour opens doors to explore SGRT as a complementary imaging modality to monitor internal changes occurring throughout radiotherapy, with no exposure costs to the patient. This is an exciting application that goes beyond its current clinical use for setup. To the best of our knowledge, this is the first time the potential of SGRT to monitor anatomical changes on treatment is proposed in paediatric radiotherapy settings.

PBT has great potential to treat children’s abdominal cancers due to its highly precise delivery and potential for fewer side effects.^9,19,34^ Nonetheless, studies have already shown how dose delivery in PBT may be greatly affected by anatomical variations.^8,9,15^ These challenges may be tackled at two key stages of the radiotherapy pathway: accounted for during treatment planning and/or adapted for during treatment delivery with IGRT information. The need to account for anatomical variations in highly conformal radiotherapy settings means that robust planning and evaluation of radiotherapy plans are essential to maintain their quality in the presence of anatomical change.^35–37^ Applying advanced planning strategies in patients predisposed to GI gas variation may help overcome the current challenges in using PBT to treat large complex tumours; findings from a previous planning study favoured IMAT in high-risk midline neuroblastomas when using standard planning techniques.^9^ IGRT strategies also help to overcome challenges caused by anatomical change by providing a method of monitoring the anatomy and triggering the need for treatment adaption accordingly. The development of adaptive radiotherapy workflows for PBT is an active area of research.^38^ In our opinion, treatment adaptation strategies that may be promising to deal with the non-deformable anatomical changes within the abdomen include online selection of the best “plan of the day” from a library of plans (optimised for different GI tract contents) or online dose restoration/full re-optimisation techniques.^39,40^ The observed correlation between internal GI gas volume and external surface variation metrics suggests there is value in exploring SGRT as a complementary paediatric imaging modality, from which easy-to-measure body surface metrics could be calculated. SGRT is a novel non-ionising imaging technique with unmet harvested potential to support safer radiotherapy treatments.^23,41^ While our findings need to be validated with clinical SGRT data, our study provides preliminary evidence of SGRT’s role in identifying timepoints with considerable GI variations, which could be used clinically to trigger more complex adaptive radiotherapy workflows. The key idea is that while SGRT would not replace CBCT imaging, it could enable a fully personalised IGRT schedule for each patient and reduce volumetric imaging to only required fractions. This would help optimising the frequency of repeat CBCT for each patient, minimising the radiation burden associated, thereby making it a promising tool in paediatric IGRT protocols. We aim to explore this clinically in the future by validating our findings with paired clinical SGRT and CBCT data in treatment position to develop a traffic light system, where SGRT is used as initial screen to trigger CBCT at each fraction.

This study expanded previous work from Lim et al^9^ where it was also noted statistically significant greater GI gas variations in anaesthetised neuroblastoma patients during radiotherapy (median 38.4%, range: 27.5 – 55.7%), compared to those without GA (11.5%, range: 7.9 – 17%). However, no correlation was established between GI gas variation and age, which contrasts results from our study where age <3.5 years was highlighted as a predictive variable. Firstly, these differences in results can be explained by the fact that our present study has a larger sample size (*n* = 21 vs *n* = 11), which likely prevented dilution of statistically significant variables. Secondly, our study used a semi-automated segmentation technique, compared to an automated-only technique used in Lim et al^9^, aiming to reduce segmentation inaccuracies in CBCTs in the presence of streak artefacts. Lastly, our present study was more comprehensive by analysing additional variations throughout radiotherapy, including body and abdominal wall separation changes.

Similarly, our findings corroborate well with Guerreiro et al^19^ where a cohort of 20 abdominal cancer patients aged 1 to 8 years old (including 11 neuroblastoma patients) displayed average GI gas changes of 99.4 ± 126.9 ml (range: −216.7 – 454.7 ml), and patient diameter changes of 0.5 ± 0.4 cm (range: −1.2 – 2.0 cm) between daily CBCT and CT. There are, however, some disparities between body contour metrics such that the numerical values are not directly comparable; our study considered the three-dimensional separation between CT and CBCT body contours, whereas Guerreiro et al^19^ assessed the separation in the anterior-posterior direction between the internal target volume centre of mass and the patient’s surface between CTs and CBCTs.

Patients without feeding tubes were observed to have greater body contour variations, which may highlight the role of feeding tubes in mitigating weight changes. The pathophysiological burdens of cancer and side effects of prior aggressive therapies could explain why weight loss is commonly reported in neuroblastoma patients.^42^ Feeding tubes are often used to manage weight loss in cancer patients,^43^ which may explain why our study observed smaller body contour variations in patients using feeding tubes. Berger et al^8^ observed that cervical cancer patients experienced weight changes between −3.1 and 1.2% throughout proton therapy, and body outline variations had a greater dosimetric impact than GI gas variations.^8^ Therefore, the link between feeding tubes and body contour variation suggests that patients without feeding tubes should be monitored more regularly when delivering very conformal radiotherapy. However, these findings were not statistically significant when excluding the planning CT from analysis so further data is required. This is likely because by excluding the pre-radiotherapy timepoint the time intervals analysed were effectively shortened, and weight loss is likely to occur over several weeks.

This study also highlighted GA as the strongest predictor for GI gas variation. This observation could be linked to the anaesthetic agent typically used in children, propofol, which targets calcium channels to induce a relaxing effect on smooth muscles lining the GI tract, including the oesophageal sphincter. The relaxation of sphincters could be a possible route for air to enter the GI system and cause variable filling in patients repeatedly exposed to anaesthetic agents.^44^ Air leaks are also a known side effect of laryngeal mask airways used for GA patients since its distal end could interfere with the oesophageal sphincter and cause gastric insufflation.^45^ Younger children are more likely to have radiotherapy under GA due to their limited compliance to lying still during radiotherapy compared to older children.^46^ Given that younger children are the target audience for PBT, their reliance on GA could indicate they will be more susceptible to anatomical variations during treatment, thereby highlighting the need for robust treatment planning and evaluation techniques for these patients. The clinical implementation of SGRT brings the opportunity of increasing the safety of dose delivery in children allowing to stop treatment in real time if movement is detected.^28^ This may bring confidence to reduce the use of GA particularly in older, more compliant children.

This study has certain limitations. First, we simulated SGRT in treatment position based on CBCT information and a six degree-of-freedom couch. This will inherently result in alignments different from those that would be achieved using standard couches and/or setup workflows with skin marks and/or planar kV imaging. Therefore, our findings need to be validated with clinical SGRT data in treatment position. Furthermore, our sample size is considered small which risks dismissing statistically significant results. Visualisation of the bowel on CBCT is very limited so our analysis was restricted to GI gas content variation. Future studies using CT-on-rails or MRI for IGRT would be of interest to investigate if our findings would apply to more complex metrics of daily bowel displacement.^47,48^ Manual editing of segmentations is prone to human errors, and the poor imaging quality of CBCT may compromise delineation accuracy. Other patient variables may be predictive of variations in contents of GI track – chemotherapy is also used in the treatment of high-risk neuroblastoma prior to radiotherapy^25^ and may be associated with GI side effects such as chemotherapy-induced enteritis and pneumatosis.^49,50^ Moving forward, this study can inform future studies investigating methods of monitoring and accounting for anatomical changes during radiotherapy. We recommend larger sample sizes and analysis of additional patient variables, including weight monitoring and details on combination treatments used, as predictors of anatomical variation.

## Conclusion

Patient variables, such as age, GA and absence of feeding tubes, were associated with greater interfractional anatomical variations. These factors may be useful to (1) inform on the selection of optimal radiotherapy modalities for each abdominal neuroblastoma patient, (2) help flag patients for robust planning and evaluation who are expected to be on a trajectory for greater interfractional anatomical variations and (3) select cases that would benefit from frequent imaging monitoring. SGRT could be a valuable tool to assist the detection of anatomical changes throughout the course of radiotherapy. The incorporation of SGRT in paediatric IGRT protocols may be useful to optimise the frequency of repeat CBCT for each patient, minimising imaging exposure.
